# Attitudes toward and Knowledge of Colorectal Cancer Screening among an Omani Adult Population Attending a Teaching Hospital

**DOI:** 10.31557/APJCP.2020.21.10.3061

**Published:** 2020-10

**Authors:** Mohammed Al-Azri, Sharouq Al-Khatri, Sathiya Murthi Panchatcharam

**Affiliations:** 1 *epartment of Family Medicine and Public Health, College of Medicine and Health Sciences, Sultan Qaboos University, Muscat, Sultanate of Oman, Oman. *; 2 *College of Medicine & Health Sciences, Sultan Qaboos University, Muscat, Oman. *; 3 *Research Section, Medical Simulation and Skills Development Centre, Oman Medical Specialty Board, Muscat, Oman. *

**Keywords:** Early detection, neoplasms, barriers, colorectal, Oman

## Abstract

**Objective:**

Colorectal cancer (CRC) is the third most commonly diagnosed cancer in Oman after breast and thyroid. Awareness regarding the availability of CRC screening services could play a major role in promoting early detection and reducing mortality rates. The aim of this study was to identify public knowledge and attitudes toward CRC screening.

**Methods:**

This cross-sectional study was conducted among 410 members of the public attending the Sultan Qaboos University Hospital in Muscat, Oman. A questionnaire was developed to assess the participants’ general knowledge, barriers and factors affecting attitudes, beliefs and behaviors regarding CRC screening.

**Results:**

A total of 410 members of the public participated from 500 invited (response rate: 82.0%). Most of the participants had not heard of CRC screening (76.3%) and were unaware of different screening methods (92.9%). The majority (93.9%) had not undergone CRC screening in Oman; however, 70.6% reported that they would be willing to do so in the future, particularly if recommended by doctors (52.7%). Barriers to screening included feeling embarrassed by the idea of a colonoscopy (73.9%), not having any symptoms of CRC (65.1%) and a fear of being diagnosed with CRC (55.6%). A bivariate analysis indicated that males demonstrated significantly more awareness of CRC screening compared to females (64.9% versus 35.1%; p = 0.004) and younger participants (<40 years of age) were significantly more aware of CRC screening compared to their older counterparts (75.3% versus 24.7%; p = 0.025).

**Conclusions:**

The majority of public in Oman showed low knowledge and awareness of CRC screening and identified several emotional barriers that might result in poor participation should screening be considered. Public education and the involvement of healthcare professionals is paramount to the implementation of a large-scale CRC screening program in Oman. In addition, addressing the sources of emotional barriers to screening is necessary.

## Introduction

Worldwide, colorectal cancer (CRC) is the third most commonly diagnosed cancer after lung and breast cancer and the fourth most common cause of cancer-related deaths after lung, breast and prostate cancer, with 1.8 million new cases in 2018 (Bray et al., 2018). By 2030, the global burden of CRC is expected to increase by 60% with an estimated 2.2 million new patients and 1.1 million deaths (Arnold et al., 2017). Generally, the incidence of CRC, particularly among men, has increased more rapidly in developing countries in comparison to developed countries as a result of the rising popularity of sedentary lifestyles that often accompany economic transition (Center et al., 2009). Nevertheless, the incidence of CRC in developed countries has recently started to stabilize or decrease, particularly in countries that have implemented national CRC screening programs (Center et al., 2009).

Overall, CRC is widely considered to be an environmental disease resulting from causative factors related to cultural, social and lifestyle-related variables (Wang et al., 2012). Dietary habits (i.e. high-fat and low-fiber diets), physical inactivity, obesity, cigarette smoking and heavy alcohol consumption all increase the risk of CRC (Willett, 2005; Rawla et al., 2019). On the other hand, certain non-modifiable risk factors have also been identified, such as aging, inflammatory bowel disease, a family history of CRC, the presence of adenomatous polyps and inherited genetic defects (Wang et al., 2012).

In the USA, the Preventive Services Task Force recommends that screening for CRC begin at the age of 50 years with a colonoscopy, with this screening method continuing on a 10-year routine basis until the age of 75 years (Bibbins-Domingo et al., 2016). In addition, fecal occult blood tests (FOBTs) should be undertaken yearly on two samples each from three consecutive bowel movements, followed by a colonoscopy in the case of positive results (David, 2006; Davis et al., 2011). Generally, a colonoscopy is considered the best screening method for both distal and proximal CRC and the combination of yearly FOBTs and colonoscopies every five years has been found to reduce CRC deaths (Wang et al., 2012; Bibbins-Domingo et al., 2016).

Located in the South-Eastern Arabian Peninsula, Oman has an estimated population of 4.6 million consisting of 2.5 million Omani nationals and 2.1 million expatriates (National Center for Statistics and Information, 2017). In Oman, CRC was ranked as the second most prevalent cancer diagnosis in 2015 among males after prostate cancer, and as the third most common among females after breast and thyroid cancer (Al-Lawati et al., 2019). Moreover, the age-standardized incidence rates of CRC were 12 and 9.1 for males and females, respectively (Al-Lawati et al., 2019). As with other Arab countries, changing consumption and lifestyle practices appear to play an important role in the etiology and increased incidence of CRC in Oman (Arafa and Farhat, 2015). Furthermore, the majority of Omani patients with CRC tend to present at more advanced stages (i.e. stages III and IV) and at a younger age (below the age of 40), with poorer prognoses (Kumar et al., 2015).

As part of national cancer control strategies, the availability of a CRC screening program plays a major role in the early detection of CRC and reduction of the mortality rate, allowing asymptomatic individuals to be identified at an early stage (Bevan and Rutter, 2018). Thus, improving public awareness of CRC-related signs and symptoms, particularly among high-risk populations, is important (Center et al., 2009). However, a previous study conducted in Oman found that the majority of members of the public were not aware of CRC symptoms (Al-Azri et al., 2019). Alarmingly, they also reported several emotional barriers to seeking early medical help, including being worried about what the doctor might find or for wasting the doctor’s time, feeling too scared or embarrassed to seek help and not feeling confident when talking about their symptoms (Al-Azri et al., 2019). Attitudes toward CRC screening are a reliable predictor of help-seeking behaviors (Rogers et al., 2018). Such attitudes are influenced by several factors including age, perceived barriers, subjective norms, socioeconomic status, educational level and previous knowledge of CRC screening (McCaffery et al., 2003; Rogers et al., 2018).

Despite their growing utilization in developed countries, the implementation of population-based CRC screening programs in Arab countries such as Oman is still inadequate due to barriers related to culture, religion, familiarity, healthcare professionals and lack of education (Arafa and Farhat, 2015). Apart from misconceptions that CRC screening is not important, Arab patients may have religious objections towards undergoing colonoscopies, particularly women, or may be reluctant due to unfamiliarity with the screening process, a general distrust of Western medicine or because they find the test to be embarrassing. Such factors have been reported to be associated with the decreased uptake of CRC screening among Arab populations in the Middle East (Qumseya et al., 2014; Arafa and Farhat, 2015; Talaat, 2015). To the best of the authors’ knowledge, no previous studies have yet been conducted in Oman to evaluate public knowledge and attitudes toward CRC screening. Therefore, the aim of this study was to identify knowledge and attitudes toward CRC screening among an adult Omani population attending a teaching hospital.

## Materials and Methods

*Setting of the study and recruitment of participants*

This cross-sectional study was conducted between December 2018 and February 2019 at the Sultan Qaboos University Hospital (SQUH), a teaching hospital in Muscat, the capital city of Oman, which receives patients referred from hospitals throughout the country. All Omani adults (aged >18 years old) who were visiting SQUH during the study period as either patients or attendees were invited to participate in the study. Seriously ill patients or those who were in pain were excluded. 

This setting was chosen as the site of the study due to the high chance of recruiting a heterogeneous sample of members of the public from different regions of Oman and also for the sake of convenience during data collection. The minimum sample size was calculated to be 400 based on predicted levels of knowledge regarding CRC screening (50%) at a precision of 5% and taking into consideration an alpha error of 5%. However, in order to allow for missing responses, a total of 500 participants were recruited. 

*Tools used to measure knowledge and attitudes toward CRC screening*

A questionnaire was developed based on the results of a literature review of similar studies conducted in other countries (McCaffery et al., 2003; Sung et al., 2008; Le et al., 2014). The questionnaire was composed of three sections. The first collected data regarding the sociodemographic characteristics of the participants, while the second measured general knowledge of CRC screening such as different methods of screening (i.e. colonoscopy or FOBT) and emotional and practical barriers to screening. The third section utilized a help-seeking behavior model to determine attitudes towards participating in CRC screening and factors affecting attitudes, beliefs and behaviors regarding screening (McCaffery et al., 2003). The latter section of the questionnaire included different questions to measure behavioral beliefs (i.e. behaviors of interest for the expected outcome); attitudes toward behavior (i.e. the positive or negative value of performing the behavior); control beliefs (i.e. factors that facilitate or hinder performance of the behavior); perceived behavioral controls (i.e. perceived ability to perform the behavior); and behavioral intentions (i.e. the probability that a person will engage in a given behavior) (McCaffery et al., 2003).

A pilot study was conducted on a sample of 50 participants in order to evaluate the comprehensibility and wording of each item and to check the reliability of the questionnaire. Feedback from the participants was subsequently incorporated into the survey. Based on a standardized items analysis, the Cronbach’s alpha coefficient of the questionnaire was 0.89. Two trained research assistants distributed the final version of the questionnaire to members of the public attending clinic waiting areas or outside rest areas at SQUH. The questionnaires were self-administered by the participants, with the research assistants collecting the questionnaires once completed.

*Statistical analysis*

Data were analyzed using the Statistical Package for the Social Sciences (SPSS), version 22 (IBM Corp., Armonk, NY, USA). Descriptive statistics were carried out on all survey items. Bivariate analyses were conducted using a chi-squared test to examine relationships between categorical variables. A p-value of <0.050 was considered the cut-off value for statistical significance. This study was approved by the Medical Research and Ethics Committee of the College of Medicine and Health Sciences at Sultan Qaboos University. The research assistants explained the purpose of the study to all potential participants and received their written informed consent prior to participation.

## Results

*Socio-demographic characteristics of participants*

A total of 410 members of the public participated in the study from 500 invited (response rate: 82.0%). The mean age was 31.5 ± 9.42 years (range: 18–55 years) and 52.0% were male. The majority (n = 249; 60.7%) were married, employed (n = 277; 67.6%), resided in the Al Batina region (n = 201; 49.0%) and had completed university and postgraduate degrees (n = 206; 50.2%). Less than a quarter (n = 78; 19.0%) had a family history of cancer (Table 1). 

*Knowledge of CRC screening*

Overall, the majority of participants (76.3%) had never heard of CRC screening and were unaware of screening methods such as home stool cards, sigmoidoscopies or colonoscopies (92.9%). While the majority (93.9%) had never undertaken CRC screening in Oman, 70.6% planned to undertake such screening in future. More than half (52.7%) were willing to undergo a colonoscopy if recommended by a doctor, with embarrassment reported as the primary reason for unwillingness to undergo this procedure (73.9%). Few participants preferred to undertake CRC screening abroad in countries such as India or Thailand (21.5%) (Table 2). 

*Reported barriers toward CRC screening*

**Figure 1:**
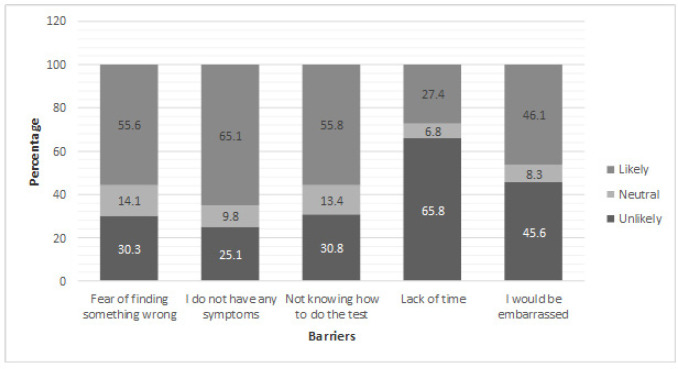
Barriers Reported to the Notion of Undergoing a Fecal Occult Blood Test as a Method of Colorectal Cancer Screening among Members of the Public Attending a Teaching Hospital in Oman (Number = 410)

Generally, participants reported barriers to undertaking FOBTs as a method of colorectal cancer screening. More than half of participants reported that they would not undergo an FOBT as a method of CRC screening as they did not have symptoms (65.1%), did not know how to perform the test (55.8%) or due to the fear of abnormal findings (55.6%). However, fewer than half of the participants thought that they would be embarrassed (46.1%) or that they wouldn’t have the time (27.4%) (Figure 1).

The majority of participants believed that it was important to be undergo regular CRC screening even without a family history of cancer (90.7%) and that this type of cancer could be cured if it was detected early via screening (89.3%). Just under half thought that CRC could be prevented by a healthy diet (48.8%) and preferred traditional or alternative medicine over modern Western medicine (49.8%). Under a quarter of the participants were of the opinion that a cancer diagnosis was essentially a death sentence (21.5%). The majority of participants reported feeling positively toward the notion of CRC screening (80.7%), but that people were nevertheless afraid of undertaking such measures (69.3%). A few members of the public felt uncomfortable with the idea of being touched by doctors during CRC screening (22.7%).

*Attitudes, beliefs and behavioral intentions regarding CRC screening*

The majority of participants were under the impression that doctors were the only ones who could request CRC screening (81.7%), but that patients should nevertheless discuss CRC screening needs with their doctors (72.9%). Half of the participants (50.0%) believed that patients were responsible for arranging CRC screening for themselves. Most agreed that they would ask their doctor if they had questions about CRC or CRC screening (92.4%) and would undertake CRC screening if told to do so by their doctor (84.6%). However, 78.0% reported that they would rely on their family or friends to take them to see the doctor if they were diagnosed with CRC. The majority stated that they would make time to undertake CRC screening even if very busy (79.5%), felt comfortable scheduling CRC screening tests (73.2%) and intended to learn more about CRC screening (71.5%) (Table 3).

According to the bivariate analysis, male participants were significantly more likely to have heard of CRC screening in comparison to female participants (64.9% versus 35.1%; p = 0.004). In addition, younger participants under the age of 40 years were significantly more likely to be aware of CRC screening compared to older participants over 40 years of age (75.3% versus 24.7%; p = 0.025) (Table 4).

**Table 1:** Sociodemographic Characteristics of Members of the Public Attending a Teaching Hospital in Oman (Number = 410)

Variables	Number	Percent
Gender		
Male	213	52.0
Female	197	48.0
Marital status		
Single	145	35.4
Married	249	60.7
Divorced	10	2.4
Widowed	6	1.5
Place of residence		
Muscat	56	13.7
A’Dhahira	64	15.6
Al Batina	201	49
A’Dakhilia	40	9.8
Al Wusta	10	2.4
A’Sharqia	25	6.1
Dhofar	1	0.2
Musandam	13	3.2
Education level		
None	7	1.7
Primary/elementary	38	9.3
High school	159	38.8
University and postgraduate	206	50.2
Occupation		
Employed	277	67.6
Unemployed	71	17.3
Student	62	15.1
Monthly income in OMR		
≤500	47	11.5
501–1,000	234	57.1
1,001–2,000	84	20.5
>2,000	45	11.0
Family history of cancer		
Yes	78	19.0
No	332	81.0

OMR, Omani riyals

**Table 2:** Knowledge of Colorectal Cancer Screening among Members of the Public Attending a Teaching Hospital in Oman (Number = 410)

Items	Number	Percent
Are you aware of CRC screening? (n = 410)		
Yes	97	23.7
No	313	76.3
Are you aware of any screening methods for colorectal cancer, such as home stool cards or a sigmoidoscopy or colonoscopy? (n = 410)		
Yes	29	7.1
No	381	92.9
Have you ever undertaken CRC screening in Oman? (n = 410)		
Yes	25	6.1
No	385	93.9
Do you plan to undergo CRC screening in the future? (n = 385)		
Yes	272	70.6
No	113	29.4
If your doctor recommends you undergo CRC screening by inserting a scope into your colon through the anus (a colonoscopy), would you agree to have this procedure performed in Oman? (n = 410)		
Yes	216	52.7
No	59	14.4
Don’t know	135	32.9
If you would not agree to have the aforementioned procedure in Oman, what would be the reason? (n = 410)		
Embarrassment	303	73.9
Religious reasons	23	5.6
Culturally unacceptable	47	11.5
Other	19	4.6
Distrust	14	3.4
Lack of medical quality	4	1
Would you prefer to undergo this procedure abroad (i.e. in India or Thailand) as opposed to in Oman? (n = 410)		
Yes	88	21.5
No	147	35.9
Don’t know	175	42.6

**Table 3:** Attitudes, Beliefs and Behavioral Intentions Regarding Colorectal Cancer Screening among Members of the Public Attending a Teaching Hospital in Oman (Number = 410)

Items	Number (percent)		
	Strongly agree/agree	I don’t know	Disagree/ strongly disagree
Behavioral beliefs			
Even if I do not have a family history of CRC, it is important to be checked regularly	372 (90.7)	33 (8.0)	5 (1.2)
CRC can be cured if it is detected early through cancer screening	366 (89.3)	38 (9.3)	6 (1.5)
I sometimes use traditional medicine to treat my health issues before trying modern Western medicine	204 (49.8)	33 (8.0)	173 (42.2)
CRC can be prevented by eating a healthy diet	200 (48.8)	120 (29.3)	90 (22.0)
Getting CRC pretty much means the person is going to die	88 (21.5)	117 (28.5)	205 (50.0)
Attitudes toward behavior			
When I think about getting screened for CRC, I feel good about it	331 (80.7)	79 (19.3)	0 (0.0)
Most people are afraid of undertaking CRC screening	284 (69.3)	86 (21.0)	40 (9.8)
I am uncomfortable about letting doctors touch my body, even if it is a CRC examination	93 (22.7)	59 (14.4)	258 (62.9)
Control beliefs			
Doctors are the only ones who can request CRC screening	335 (81.7)	47 (11.5)	28 (6.8)
People should talk to their doctors about what CRC screening they need	299 (72.9)	67 (16.3)	44 (10.7)
People are in charge of arranging their own CRC screening	205 (50.0)	158 (38.5)	47 (11.5)
Perceived behavioral controls			
If I had questions about CRC or CRC screening, I would talk to my doctor	379 (92.4)	23 (5.6)	8 (2.0)
If a doctor told me to get CRC screening, I would do it	347 (84.6)	47 (11.5)	16 (3.9)
I would rely on my family or close friends to take me to see a doctor if I had CRC	320 (78.0)	37 (9.0)	53 (12.9)
Behavioral intentions			
Even if I were very busy, I would make time for CRC screening	326 (79.5)	77 (18.8)	7 (1.7)
I feel comfortable scheduling CRC screening tests	300 (73.2)	89 (21.7)	21 (5.1)
I intend to learn new information about CRC screening	293 (71.5)	95 (23.2)	22 (5.4)

CRC, colorectal cancer

**Table 4:** Associations between Demographic Characteristics and Awareness of Cancer Screening among Members of the Public Attending a Teaching Hospital in Oman (Number = 410)

Variables	Awareness of CRC screening, Number (percent)		p-value
	No (n = 313)	Yes (n = 97)	
Gender			
Male	150 (47.9)	63 (64.9)	0.004*
Female	163 (52.1)	34 (35.1)	
Age group (years)			
<25	78 (24.9)	34 (35.1)	0.025*
25–40	175 (55.9)	39 (40.2)	
>40	60 (14.6)	24 (24.7)	
Marital status			
Single	110 (35.1)	35 (36.1)	0.903
Married	203 (64.9)	62 (63.9)	
Education level			
Primary/elementary	35 (11.2)	10 (10.3)	0.948
High school	122 (39.0)	37 (38.1)	
University and postgraduate	156 (49.8)	50 (51.5)	
Occupation			
Employed	172 (55.0)	54 (55.7)	0.908
Unemployed	141 (45.0)	43 (44.3)	
Family history of cancer			
Yes	58 (18.5)	20 (20.6)	0.658
No	255 (81.5)	77 (79.4)	

CRC, colorectal cancer; *Significant at p <0.050.

## Discussion

To the best of the authors’ knowledge, this is the first study conducted in Oman to explore knowledge and attitudes toward CRC screening among adults of the general population. Although more than half of the participants reported a willingness to undergo CRC screening, the majority were not aware of such screening and had never undertaken CRC screening measures before. Previous studies conducted in both Western and Arab countries have reported similar findings (Taskila et al., 2009; Gimeno-García et al., 2011; Qumseya et al., 2014; Galal et al., 2016). Poor knowledge or intentions of participating in CRC screening have been associated with various factors, including socioeconomic status, ethnicity, age and gender (Weller et al., 2007; Qumseya et al., 2014; Le et al., 2014). Lack of knowledge of the importance of CRC screening and limited literacy have been identified as important barriers to participation in screening programs in Islamic countries (Bidouei et al., 2014; Galal et al., 2016). Indeed, as in other Arab countries, lack of knowledge regarding the availability of CRC screening in Oman may also contribute to low awareness of its importance or necessity (Galal et al., 2016; Fadhil and Al Hammod, 2018). People who are unaware of the importance of CRC screening frequently demonstrate poor attitudes toward and limited intentions of participating in cancer screening measures (McCaffery et al., 2003; Berkowitz et al., 2008). Thus, development of a national screening program is an important strategy to increase public knowledge and participation in CRC screening (Wee et al., 2005; Galal et al., 2016).

While the majority of participants in the present study were willing to participate in CRC screening if recommended by doctors, they were nevertheless unaware of either the importance or availability of CRC screening measures in Oman. A previous study conducted in Oman similarly found that more than half of the participants would have a colonoscopy if doctors advised them to do so (Al-Azri et al., 2019). Furthermore, the majority of participants in the present study were of the belief that only doctors had the authority to request CRC screening measures. Lack of awareness concerning CRC screening has been linked to limited recommendations for CRC screening on the part of healthcare practitioners and medical recommendations from healthcare practitioners are a strong motivator and predictor for screening participation (Janz et al., 2003).

Previous studies conducted in Oman and other Arab countries have noted that the majority of doctors and nurses do not encourage patients to take part in CRC screening as they themselves are not knowledgeable with regards to the recommended frequency of CRC screening or the importance of early CRC screening for high-risk patients (Galal et al., 2016; Muliira et al., 2016). In Palestine, only 14% of participants stated that their doctors had informed them of CRC screening measures (Qumseya et al., 2014). Lack of continuity of care and not having a regular care provider has been associated with low participation in CRC screening measures (Harewood et al., 2002). 

In Oman and other Middle Eastern countries, local healthcare systems do not support continuity of care as patients are often seen by any doctor available whether they attend governmental or private health institutions (Mohammed Al-Azri et al., 2014; Saleh et al., 2015). Thus, improving knowledge of the importance of CRC screening and continuity of care among doctors and healthcare practitioners in Oman should be considered (Muliira et al., 2016). Unfortunately, many doctors practicing in local health centers in Oman lack the time to educate their patients regarding screening measures, especially if they themselves are unaware of official recommendations. Thus, national guidelines for CRC screening should be published at the primary and secondary healthcare levels to increase awareness of CRC screening and risk factors among healthcare practitioners.

Other factors found to contribute to a refusal to participate in CRC screening measures are emotional barriers, such as embarrassment or anxiety concerning the procedure. A previous study conducted in Oman found that the main reasons for refusing a colonoscopy, even if advised by doctors, were embarrassment, lack of trust in the healthcare provider and religious or cultural beliefs, with around 39% of participants preferring to undertake colonoscopy examinations abroad (Al-Azri et al., 2019). Religious objections and cultural barriers towards colonoscopies are often reported as reasons for refusing to participate in screening, especially among Muslim women living in conservative societies; as such, ensuring female patients have the option of being screened by a doctor of the same gender might encourage participation in CRC screening Oman (Qumseya et al., 2014; Arafa and Farhat, 2015; Attum and Shamoon, 2018). On the other hand, even in Western countries, studies have shown that women are less likely than men to agree to be tested by flexible sigmoidoscopy, although other researchers have reported no significant differences according to gender (Janz et al., 2003; Meissner et al., 2006; Shokar et al., 2009).

Indeed, in the present study, male participants were significantly more likely to demonstrate awareness of CRC screening compared to their female counterparts. Omani women were more likely than men to report barriers to seeking early medical help for possible CRC symptoms due to fear, difficulties arranging transport and concern regarding what the doctor might find (Al-Azri et al., 2019). Similarly, in Saudi Arabia, women were reported to be significantly less likely to participate in CRC screening (Galal et al., 2016). Thus, should future measures to encourage participation in CRC screening be undertaken in Oman, special care should be made to ensure that education or awareness efforts are targeted equally to women.

In addition, younger participants in the present study were significantly more likely to be aware of CRC screening compared to older participants. This indicates that middle-aged and elderly patients in Oman may be at greater risk of missing out on early CRC screening measures and potentially being diagnosed at a later stage. Generally, older people tend to perceive themselves as having a lower risk of CRC, a fact which could go some way toward explaining their apparent lack of concern in CRC screening (Taskila et al., 2009). In addition, the formal education system in Oman was implemented only 50 years ago, which might explain why older participants were less aware of CRC screening compared to younger individuals. Less educated people are also reportedly less likely to receive information or counselling from their doctors regarding CRC screening, a factor which may contribute to low awareness (Wee et al., 2005).

Many participants in the present study reported fearing CRC screening or receiving abnormal results from cancer screening. Such fears could lead to poor participation should a CRC screening program be established in Oman in future. Perceptions of social stigma and feelings of fatalism regarding a cancer diagnosis also contribute to low acceptance of CRC screening (Al-Azri et al., 2014; Palmer et al., 2015). A previous study conducted in Oman indicated that a diagnosis of cancer often has a devastating psychosocial impact on the individual, including anxiety regarding treatment side-effects and the thought of dying (Al-Azri et al., 2014). Another study from Oman also found that most barriers to seeking early medical help for CRC symptoms were emotional, including fear of the results of screening (Galal et al., 2016). Initiatives to increase public knowledge of the importance of CRC screening are necessary, perhaps by way of a national education program implemented via traditional media (such as radio, television and newspapers) as well as on social media and the distribution of leaflets and posters in the government hospitals and at local health centers in different regions of Oman.

*Limitations*

Limitations of the current study include the fact that it was conducted among members of the public attending a single teaching hospital, potentially affecting the generalizability of the results. However, patients or attendees visiting SQUH often reside in other regions of Oman, a fact which could minimize this bias. Second, although the questionnaire used to collect data was adapted from previous literature, a pilot study revealed that it demonstrated high reliability. Nevertheless, a larger national study involving individuals from all regions of Oman is required for better representative sampling. Third, a subset of the participants reported a family history of cancer, which might have resulted in report bias. 

In conclusion, many members of the Omani public were not aware of or had previously taken part in CRC screening, although the majority reported a willingness to undertake such screening in future. Policy-makers should develop strategies to increase of CRC and accompanying screening measures among the general population. A national screening program for CRC should be implemented to promote screening among individuals aged 40 years and older, particularly in high-risk groups such as those with a family history of CRC. In addition, identifying and addressing emotional barriers and negative attitudes toward CRC screening is necessary, particularly for women and older patients. Finally, further exploratory research is needed to identify the causes of and solutions for emotional barriers toward CRC screening in Oman. 
